# Special Considerations for Endoscopists on PEG Indications in Older Patients

**DOI:** 10.5402/2012/607149

**Published:** 2012-11-25

**Authors:** Fabrizio Cardin

**Affiliations:** Geriatric Surgery Unit, Geriatric Department, Padova University and General Hospital, Via Giustiniani 1, 35100 Padova, Italy

## Abstract

Undernutrition in frail elderly people is a pathological condition that needs to be recognized and addressed early. Neurological dysphagia is among the most frequent causes of this condition in the elderly but should be considered a terminal event in Alzheimer-type dementias. Tube feeding is an important resource for facilitating metabolic recovery in cachectic patients and is particularly successful in “bridging” and stabilizing therapies prior to major treatment able to cure the patient. Clinical management of tube feeding in “incurable” conditions is complex and becomes part of the palliative care and comfort provided in the terminal stages of illness. Non-specialized physicians are often unfamiliar with the theory and practice of end-of-life interventions, and the resulting decisions are in many cases actually contrary to patient comfort. These problems deserve to be more carefully addressed when the patient is unable to cooperate or express his/her preferences and needs. The success of percutaneous endoscopic gastrostomy has led to increasingly frequent referrals for placement in critically ill elderly patients. Endoscopists therefore become a key figure in stimulating rational, correct treatment of these patients.

Legal and ethical issues are becoming an increasing part of the work of endoscopists. This is due mainly to the risk of procedure-related complications and, more rarely, to cases where the proposed intervention may be of no benefit to the patient and consequently deemed “futile” [1, 2]. Since endoscopy is traditionally considered a “minimally invasive” procedure, adopted when invasive surgery is not feasible, ethical problems are relatively rare. The ability to recognize the “futility” of a treatment does, however, require some “interface” knowledge in the fields of oncology, cardiology, and anaesthesia, which are often beyond the endoscopist's standard cultural and educational background. A procedure can be considered futile when it is unlikely to produce the desired outcome or, if it does, to be of no benefit to the treated patient [3].

Ethically and legally speaking, percutaneous endoscopic gastrostomy (PEG) placement requires close assessment of the patient's clinical conditions and prognosis [4, 5], which may disorient providers accustomed to concentrating on the technical aspects of the procedure. Likewise, Scott suggested that endoscopists ought to adopt a more comprehensive approach to PEG placement, particularly in elderly or critical patients [6]. Placement affects all subsequent ethical decisions by virtue of the very ethical differences between nonintervention and withdrawal of an ongoing treatment, in this case artificial tube feeding [7, 8]. Endoscopists who attend solely to the technicalities of the procedure without adequate clinical assessment and knowledge may be accused of “ageism” which essentially consists of making errors due to age-related discrimination, as authoritatively reported elsewhere in the literature [9]. It is therefore worth making a few considerations on the issue of PEG placement with a view to building an interface relationship between endoscopy and geriatrics.

Endoscopists will probably play an increasingly key role given the growing elderly population admitted to endoscopy (resulting in all specialist services and wards being overcrowded by older adults) [10, 11]. In many cases, the attending provider is not a geriatrician (with adequate management knowledge of the chronic degenerative pathologies afflicting older adults) but another doctor without such expertise. Elderly people are increasingly referred for PEG placement by ENT specialists, surgeons, orthopaedists, intensivists, or general practitioners, more involved in resolving the dysphagia or aspiration pneumonia in its acute stage than in assessing how the requested intervention will influence the elderly patient's life expectancy [12]. Getting endoscopists to improve their knowledge on treating dysphagia in geriatric patients may ensure better control “at source” of how this important therapeutic tool is used.

Dysphagia is common among the elderly, causing repercussions on nutritional state and drug administration, with increased risk of pneumonia and a poorer quality of life. Prevalence rates are as high as 30–40% among institutionalized elderly people [13]. The use of videofluoroscopy in these patients, some of whom are symptom-free, has identified anomalies in the swallowing process in 63% of cases [14]. Dysphagic disorders are associated with a worse prognosis and higher mortality at six months, prompting the need for early identification in order to rapidly intervene to prevent the onset of malnutrition. In many cases, however, these patients are affected by severe physical and/or mental disability, making it difficult to perform an adequate diagnostic assessment and begin appropriate physiotherapeutic treatment. At present, 25 million people throughout the world are affected by dementia, with 4/6 million new cases per year [15]. The incidence of dementia in Europe rises from 1 case per 1000 population per year at age 65 years to 8 per 1000 after 85 years [16, 17]. Alzheimer's disease accounts for 50–60% of all forms of dementia [15], and, considering the demographic revolution in progress, the number of people affected by Alzheimer's is set to increase threefold in the next fifty years. In 1907, Alois Alzheimer characterized the disease for the first time, describing the “slowing progressive decrease in body weight” as a characteristic symptom. In fact weight loss can be higher in Alzheimer's sufferers than in patients with cancer or heart failure [18].

As the general population has aged, so has there been a rise in the number of hospital admissions with malnutrition and dysphagia as one component of a more complex clinical picture, and it is difficult to make a prognosis solely on the basis of specific biochemical parameters [19]. Rather than assess individual chronic pathologies, geriatricians have long standardized their approach to the elderly by opting for a comprehensive geriatric assessment, which essentially evaluates life expectancy and residual autonomy on the basis of existing pathologies [20, 21]. Global assessment is undoubtedly a very useful method of measuring swallowing impairment which may be reversible in some cases, but part of a terminal picture in others [22]. In one cohort study on residents of 22 nursing homes affected by advanced dementia, over half of the residents were reported to have died within 18 months of admission, mainly from pneumonia, fever, and feeding difficulties. Of these, approximately 41% had undergone important procedures in the final three years of life, as admission to an emergency care unit, parenteral therapy, or tube feeding [23]. An interesting study by Wolfson reported that life expectancy in a sample of 821 patients affected by dementia, the majority of them women with Alzheimer's, was 3.3 years (95% CI 2.7–4) from time of onset. He also noted that, as might be expected, the onset of dementia at a younger age was associated with greater life expectancy [24].

In older adults, however, many clinical manifestations do not fit into clearly defined nosological categories and making a prognosis is one of the most difficult tasks that physicians have to perform, with no shortage of mistakes [25]. The general approach to dysphagia in the elderly is based on experience with stroke patients, in whom early artificial nutrition is recommended to maintain metabolic status. One historic study on stroke patients demonstrated that early tube feeding is associated with an absolute risk of death of 6% and an approximately 2% reduction in adverse reactions. PEG placement is requested in only 28% of stroke patients as a means of stabilizing assisted feeding support, which should be implemented after a patient has proved unable to feed him/herself after 2-3 weeks using a nasogastric tube [26, 27].

In tube feeding, PEG placement can clearly be considered a successful technique that is relatively easy to perform and may be part of the experience of any endoscopist [28, 29]. Morbidity rates are reported to be 17%, with severe events occurring in around 3% of cases [30]; feasibility stands at 95–98% [31–33]. Accordingly, since 1980, there has been a major increase in PEG placements, with numbers doubling between 1988 and 1995 in patients aged over 65 in the USA [34], and even more pronounced trends being observed in British hospitals. The placement rate in The Netherlands was found to be 3.4 per 100 patients/year, and approximately 184,000 PEG placements were made in Germany [35, 36]. Epidemiological assessments have, however, revealed critical factors resulting from disparity of use, with rates varying in different parts of the same country from 6 to 20% of nursing home residents. PEG placement rates tend, for example, to be influenced by the location of the nursing home to which patients with dysphagia are admitted (rural versus urban). This is linked to the presence of nearby surgical services, presuming that the use of the technique is related more to provider/consumer ratios or cultural approaches in the various areas than to objective clinical assessments [37].

The success of the technique has led to the publication of various case series which have reported a 30-day mortality rate of around 25%, in contrast to the success rates recorded at time of placement and to intuitive evidence that improving nutrition leads to better survival. In addition, the mean survival of patients who die during hospitalization for PEG insertion was less than 26 days [38]. Dharmarajan et al.'s findings showed a survival of 9.5 months for patients younger than 65 years, 7.5 months for those between 65 and 74, and less than six months for older patients [39]. It is interesting to compare these rates with the now historic 30-day mortality rates of 15% for another endoscopic procedure: biliary prosthesis insertion [40]. The verification process implemented to address the findings led, amongst other things, to the adoption of new stent models and biliary endoscopic techniques. Percutaneous gastrostomy does not seem to have been subject to the same level of criticism for its 30-day mortality.

Interpretation of mortality data should be based on epidemiological statistics. These show that the over-65 age group exhibits the highest rise in PEG insertion rates and that the patients aged over 75 who most frequently receive tube feeding are the ones with greater comorbidities. The higher increase has been shown to be linked to greater use of this technique in hospital settings, with no differences between profit and not-for-profit facilities. The mean recorded increase was 1.4/10,000 admissions for patients aged under 65 years, 14 per 10,000 for 65–74-year-old patients, and almost 31 per 10,000 for those aged over 75. Use of the Elixhauser comorbidity scale proved that the more severe patients were the tube-fed ones [41].

On associating these rates with underlying pathology, dysphagic patients with dementia appear to have the lowest post-PEG placement survival rate, compared to patients with oropharyngeal cancer, stroke, or other neurological pathologies, as motor neurone disease or traumatic brain injury [42]. Patients affected by Alzheimer's disease are at higher risk of malnutrition; involuntary weight loss is present in approximately 40% of patients and can present at any stage in the disease. Inadequate food and energy intake result from various factors, including anorexia, depression, apraxia, and even increased energy expenditure due to hyperactivity. Eating disorders are present in 22–56% of cases, according to the severity of the pathology. Assessment of eating disorders is particularly important in moderate to severe patients since specific care prevents malnutrition and averts the adoption of artificial feeding techniques.

The residual life of these patients is also affected by the long-term complications of PEG, which are more prevalent than perioperative adversities, with rates reaching 27% for stomal discharge and pain and 16% for local infection at the stoma site [43, 44]. When in inexperienced hands, feeding tube utilization can cause occlusion-related problems and general care expenditure may increase as a result of more frequent recourse to emergency services for tube dislodgement [45]. Very elderly patients are clearly functionally impaired and the very need for them and their families to be adequately equipped for purpose can cause problems, as reported by McNamara et al. and Pancorbo-Hidalgo et al. Furthermore, in the long term, systemic symptoms may be experienced, as nausea, vomiting, and abdominal alterations in around 40% of patients, and food regurgitation and pneumonia in 28 and 34%, respectively. During the enteral nutrition period, a mean rate of approximately 4.1 ± 4.7 complications of various types was recorded per patient. A necessary commitment was also made to avoiding hydroelectrolyte imbalance [46, 47].

Numerous studies have assessed prognostic factors for early mortality with PEG, to aid those responsible for deciding on PEG placement. These studies have highly heterogeneous designs, making it very difficult to adopt a systematic approach. We were interested in assessing which factors recurred most frequently and about half of the thirteen considered studies reported aspiration pneumonia, hypoalbuminemia, and older age [39, 48–57]. The admission setting also seems to be important because in the study by Lang et al., patients from nursing homes were found to have a better prognosis than those whose PEG was inserted during an acute admission [58]. Only Gaines et al. reported that dementia did not appear to predict a worse outcome [59]. Paradoxically, many of the above-mentioned factors are often used to refer patients for tube feeding.

Essentially, however, overall deterioration is the most significant factor in predicting post-PEG survival, as rated by the Charlson index, which is one of the most widely used scales in global geriatric assessment [60–62]. Only one study indicated the probability rate for PEG withdrawal in geriatric patients. This was reported to be 22 out of 59 patients after one year but it should, however, be noted that the number of patients with dementia in this case series was 15.

The geriatric concept of frailty, which basically describes an elderly person's inability to adapt and to tolerate stress, may affect the presence of esophagogastric lesions [63]. It is, in any event clearly a dynamic status [64], and the timing of placement with respect to acute decompensation is another factor affecting the probability of post-PEG survival.

As regards the source of patients admitted to PEG, patients treated during acute hospitalization had a poorer prognosis than those coming from their normal place of residence [65]. Moreover, findings have shown that patients undergoing PEG placement later in their hospital stay have a higher life expectancy. Paradoxically, therefore, assisted feeding appears to be more the means for maintaining the clinical equilibrium of the elderly patient than the means for achieving it. In the study by Abuksis et al., postendoscopy mortality was in fact lower among elderly undergoing placement 30 days after referral, according to an intention-to-treat analysis [66]. This observation has led to a practical recommendation to delay endoscopy and to adopt a nasogastric tube, implementing a sort of “grace period” to help prevent mortality after PEG insertion. This recommendation was corroborated by the experience of Smith et al. [52].

What appears to be most important in the decision-making process is failure by the various carers to get the elderly person to eat. However, a study conducted in Italy on 40 elderly people affected by Alzheimer's disease with severe cognitive deficit and comorbidity showed that, despite being time consuming, the adoption of correct stimulation techniques designed to preserve natural feeding left metabolic balance unaltered over time. In this study almost half of the patients were judged to be malnourished based on body mass index, albumin transferrin, cholesterol, and haemoglobin index. The techniques to preserve natural feeding consisted of environmental changes to encourage concentration on eating, the definition of a quality diet with a consistency acceptable to the patient, and nutritional supplements. The number of malnourished patients fell by one quarter, with a significant increase in albumin and positive variations in other parameters [67]. However, the ratio between adequate calorie intake and weight changes is critical, particularly in patients with Alzheimer's disease. Even with an adequate intake of calories, protein, and vitamins, these patients can continue to lose weight. What does influence them is their caregiver and their caregiver's awareness of and compliance with the indications provided by their attending doctor and physiotherapist.

The characterization and assessment of dysphagia is probably one of the most influential factors in the outcome of feeding intervention. It is not easy to objectively assess swallowing ability in the elderly with dementia since their lack of cooperation and their removal from their care setting make it difficult to apply standard techniques. Consequently, simple practical bedside methods have become widespread. Some of these require specific training in order to be carried out by providers who are not speech therapists, but they do generally seem to lack the necessary sensitivity. The potential for conducting videofluoroscopy as the gold standard in poorly collaborating patients is also limited. The most widely used assessment method is the gag reflex which, when intact, may be protective against the need for enteral feeding even in the long term [68, 69]. Salassa suggested a helpful classification combining the assessment of functionality with the impact of impaired function. This classification is divided into the following 5 stages:

- normal function with minimal dietary changes, regular meals but symptomatic episodes in the presence of reflux, difficulty in chewing, and clearing the mouth after swallowing;
- abnormal swallowing which is recovered following significant changes in diet or prolonged eating times, with episodes of coughing that avoid infectious complications; weight is kept constant through this commitment to care;
- decompensated dysphagia due to marked weight loss over a six-month period and presence of coughing during almost all meals; the patient is considered to be at risk but no respiratory complications have yet appeared;
- presence of dysphagia inducing significant weight loss and respiratory complications; oral feeding only allowed if antiaspiration rules and monitoring are strictly followed, but this does make it difficult to maintain the necessary calorie intake;
- complete swallowing failure with inability to swallow anything safely.

This assessment scale is proposed because of its simplicity and better chance of establishing the best time to start artificial feeding support [70].

An interesting observation was made by Kitamura et al. who calculated the delay in swallowing reflex following laryngeal stimulation by assessing normal swallowing reflex within 3 seconds of instillation of 0.4 mL and 0.2 mL of distilled water through a 4-5 Fr small nasal catheter. He used this to show that the onset of aspiration pneumonia was determined by degree of dysphagia associated with the presence of esophagitis, but not hiatal hernia diagnosed during placement endoscopy in patients with PEG. Based on this test, he proposed a score to predict airway aspiration designed to better monitor PEG patients at risk [71]. This observation, albeit limited by the small sample size, turns our attention to one of the most frequent reasons for requesting PEG placement, that is, to prevent respiratory complications secondary to swallowing impairment. Aspiration pneumonia has been reported to be present in 3% of the nursing home population aged over 65. Data were evaluated on 102,842 patients, and, on analysing 55 independent variables, tube feeding was shown to have a role in bringing about respiratory complications, with an odds ratio of 1.73. This puts it in the fourth place in the study by Langmore et al., after suctioning, chronic obstructive pulmonary disease, and congestive heart failure [72].

The association between tube feeding and risk of infection in the elderly has been assessed in two other studies. In one, designed to examine the clinical factors influencing the presence of antibiotic-resistant bacterial infections in institutionalized elderly by multivariate analysis, tube feeding had an OR of 3 and was found to be significant, together with previous use of antibiotics [73]. In the other study, exploring pneumonia in frail elderly people, oral flora was compared in three groups of nursing home patients according to feeding method (oral, nasogastric tube, and PEG), showing that PEG facilitated colonization by pathogenic oral flora such as *Pseudomonas aeruginosa*, *Klebsiella*, and *Proteus*. This finding suggests that, besides reconsidering the protective potential of PEG towards aspiration pneumonia, careful hygiene and oral care are also needed in patients fitted with the device [74]. Moreover, assisted feeding does not seem to indirectly protect against infections since it fails to bring about any improvement in metabolic and anthropometric variables in elderly with permanent tube feeding [59].

At least six guidelines have been published to address the contrasting evidence on the use of PEG in elderly people with dementia and to provide management proposals. The Mayo Clinic guideline [75] starts from the consideration that anorexia accompanies the most severe pathologies through a cytokine-mediated fall in appetite and that a calorie intake of 1000–1200 kcal is sufficient for an elderly person, partly due to the cascade of metabolic changes occurring during starvation processes. Clinical observations have shown that ill persons can survive for approximately two weeks if completely deprived of fluids and nutrients. Conscious people with terminal illness do not complain of hunger or thirst, which can be satisfied with limited intake of food or fluids or even by moistening the oral mucosa, all of which appear to be accompanied by a reduction in suffering. In addition, this guideline acknowledged that aspiration of foodstuffs during tube feeding was common and that it did not improve nutritional status.

In the terminal illness care setting, these observations prompt the need for multidisciplinary support for relatives and families. The gastroenterologist can review and obtain consent for the procedure, preserving patients with impaired cognition from inappropriate decisions and relying on the legally binding or informal choice of the most appropriate surrogate decision maker for the patient. The specialist should also be mindful of the tendency among normal reference general medicine providers and family doctors to overestimate the benefits of PEG in patients with advanced dementia. Moreover, the admission requirements of many nursing homes indifferently request the presence of preplaced tube feeding, with the consequent risk of patients undergoing various forms of restraint. UK guidelines [76], referring to more general use of PEG and the technical aspects of placement and postcare, report that the problem of undernutrition is very common in hospitals and influences patients' clinical course. Given that there are few biological and physical surrogates to define malnutrition, particularly in long-term bedridden, uncooperative patients, the guideline suggests that providers should maintain a high level of clinical suspicion. The guideline also indicates that it would be useful to provide general rules of orientation because if PEG placement is programmed at a terminal stage of the disease, ethical considerations demand that it should only be performed when it serves to alleviate any symptoms and not necessarily to prolong survival. When in doubt, a limited test period can be implemented, using less definitive, invasive means, although 25% of nasogastric tubes do subsequently fall or are pulled out.

The general objective is to limit bad patient selection considered to be the cause of very high patient mortality a few weeks after the procedure. In an extensive review, Angus and Burakoff [77] usefully summarized the correct indications for PEG insertion into four points: (1) esophageal obstruction; (2) neurological aetiology of dysphagia without obstruction (cerebrovascular accident, pseudobulbar palsy); (3) prolonged refusal to eat without evidence of terminal disease; (4) nutritional supplementation in patients undergoing radiochemotherapy. In all cases, however, assessment hinges on the principle of induced and expected benefit, thus ruling out aspects of anorexia-cachexia syndrome, and on the fact that intervention in the course of acute illness is the main cause of adverse events after PEG.

Significant comparative data have also been reported on the use of PEG versus the small-bore feeding tube. Clinical outcome appears to be similar, and there are similar incidences of aspiration pneumonia beyond the first 14 days, with comparable mortality rates two weeks after insertion. In agitated patients, PEG seems to be better tolerated than nasogastric access, although the risk of extubation does remain but can be reduced with abdominal covers.

To tie up these concepts, one of the first ethical assessments of PEG, published in 1997 in The Lancet [78], advanced the principle that if a given procedure produces positive results (as the prolongation of survival) but is associated with unfavourable consequences (as the rise in symptoms secondary to progression of the existing disease), the overall benefit becomes uncertain, in which case the principle of respecting the patient's independent decision comes into play. Counselling (informative follow through) is also differentiated from the directive position. In bedridden patients with Alzheimer's disease, it is suggested that the physician should assess residual aspects of quality of life that are destined to worsen as the disease progresses and weigh up the alternatives to tube feeding and the incidence of induced complications in the given circumstances. Where there is no valid decision-making assistance, including family support, the recommended strategy is to seek the intervention of an ethics committee. Within this ethical orientation, the gastroenterologist is explicitly assigned the role of key player and no longer simply makes a technical contribution to decisions made by others. The advantage of full gastroenterologist involvement is, however, also technical since the guidelines of the American Gastroenterological Association acknowledge [79] that this seems to have a beneficial effect, by reducing complications compared to other endoscopic specialists. The need for a nutrition support team composed of dieticians, nurses, and pharmacists with experience in nutritional support is also suggested to manage patients completely and cost-effectively. This lowers complications, often reduces the need for aggressive approaches, and provides the best support for challenging patients [80]. By way of a summary, the following directives can be drawn from the analysed guidelines:

- PEG placement is recommended for patients who can benefit from it for at least 30 days;
- PEG should not be included in management plans for patients with Alzheimer's disease;
- PEG placement should not be offered in the absence of proven benefit;
- where there are any doubts about assisted feeding, placement of a nasogastric tube can be considered for a limited trial period;
- PEG placement cannot be programmed for unstable patients;
- artificial nutrition is not recommended in patients with end-stage dementia.

Unfortunately no controlled studies have been published for ethical reasons, and any directives provided are chiefly based on observational and retrospective studies. Studies designed to define the usefulness of implementing pragmatic directives to improve patient survival have instead shown contrasting data. One study compared outcomes in two different periods in the same hospital, before and after an audit on PEG use. It showed, on the one hand, the difficulty of pathway standardization and, on the other, the difficulty of controlling the exponential rise over the years in PEG placement procedures in the absence of any influence on capacity to select the patients with the best survival at thirty days after procedure [81]. The second study started from the basic consideration that demented patients' inability to feed themselves may stem from anorexia rather than dysphagia, in which case the process of assisted feeding may be the equivalent of forced feeding. In the absence of clear experimental evidence, the endoscopist, who does not only have a technical role, must identify the most effective medical intervention for these patients rather than the one driven by care or administrative expedience.

On the basis of the above considerations and the historic finding of higher mortality in tube-fed demented patients, pragmatic indications were drawn up and implemented in a British hospital and another hospital was selected for control purposes. While there was a reduction in the number of endoscopy procedures in the study hospital, there was also a trend towards a fall in the mortality rate, but not a significant one [82]. In both cases, reasoned adoption of PEG based on set directives yielded useful but not significant trends in improved performance. As stated above, these guidelines lack randomized data comparing the long-term results of tube feeding versus maintenance of oral feeding based solely on residual swallowing ability. Various observational studies have been published, of which we have identified eleven issued between 1997 and 2008. While not directly comparable due to differences in design, three of them did report positive outcomes for maintenance of natural feeding [83–85].

A study by Tokuda and Koketsu [86] compared the mortality of 106 patients, of whom 15% had received tube feeding, with factors predicting mortality during the index hospitalization. Findings showed that tube feeding alone was associated with higher mortality compared to other factors, as the presence of pneumonia, a history of hip fracture, and older age. In another comparative assessment conducted retrospectively over 2 years on the nursing records of 41 patients with advanced dementia referred for PEG, which had only been performed in 23 cases, survival was the same in the two groups (59 versus 60 days) and there were complications in 4.3% of patients admitted to PEG [87]. Interestingly, comparative assessment in another group of 57 patients with enteral feeding versus a numerous control group [88] showed that while some biohumoural parameters, such as haemoglobin, lymphocyte count, renal function tests, osmolarity, and serum proteins, improved in the treated group, mortality was also higher in this group (42% versus 27%; *P* > 0.05), with a higher number of complications, probably due to the higher number of comorbidities in these patients. An American observational study on a single group of 150 patients treated with PEG (minimum age 60 years) and constantly monitored for 14 months to assess functional status, cognitive status, comorbidity, and quality of life showed no substantial improvement in these parameters. The authors concluded that randomized trials of PEG feeding compared to alternative methods were warranted, an opinion undoubtedly shared by many [89].

Only three reports have highlighted positive aspects of feeding tubes in the elderly. One study conducted in Turkey, in which the average patient age was 55.94 ± 16.14 years old, with patients affected by different neurological pathologies (only 11% with dementia), showed an improvement after PEG placement in all nutritional indexes considered (body mass index, midupper arm circumferences, and triceps skin-fold thickness). The study was not comparative, and the 85 studied patients had 14 early complications and 18 late complications; total mortality was 37% in the observation period from March 1999 to September 2004 [90].

By analysing an administrative reimbursement tool and comparing the data of two US states in 1993 and 1994 in which patients with progressive feeding dependence were tube fed, survival at one year was 39% compared to 50% in patients without assisted feeding. Hence, the procedure was found to be beneficial but did present very high mortality rates [91].

A Japanese study comparing three groups of bedridden elderly fed orally, parenterally and enterally, showed a survival advantage for PEG (survival of 2, 8, and 23 months, resp.) [92]. One study reported on the positive withdrawal of PEG (26%), but this was linked to patient characteristics (younger age) and better renal functioning rather than to tube feeding bringing about an improvement [49].

The question of PEG placement in the elderly is influenced not only by metabolic factors but also by the organizational-management concerns of the admitting institutions and patients' relatives. Management uncertainties and directives that are uninformed or fail to comply with current guidelines clearly result in a wide variability of tube feeding utilization rates in institutionalized people. In Canada, where clinical directives on nutritional support were defined long ago but refer to intensive care units and therefore to a different setting from the one considered in this paper, findings have shown poor provider compliance with preset operative guidelines in this field [93]. This may be thought to depend on the difficulty of implementing audits and guidelines in everyday practice, but statistical analyses have yielded factors linked to different clinical conditions of patients and type of hospital, that is, university versus general, and the sex of the patient and type of admission, that is, medical versus surgical.

A survey carried out in nine North American states showed variations in PEG placement of between 7.5% in Maine and 40% in Mississippi, without evidence of influential medical factors, whereas differences in legislation among states and their interpretation can carry weight and affect medical choices [94].

Five studies have provided some insight into the structural features of facilities with the highest tendency to utilize tube feeding. In a study on an extensive Veteran Health Administration database, carried out during the 1990s, there was found to be a rising trend among demented patients aged over 60 years as soon as the PEG technique was introduced. The percentage of placements subsequently fell, although racial differences were found in their case series. Being Afro-American and demented led to a relative risk of 1.65 of undergoing tube feeding. This risk factor recurs in other studies. On applying stepwise logistic regression analysis, predictors of PEG placement in demented patients were again found to be belonging to the Afro-American race (OR 9.4 95% CI 2.1–43.299) and being a nursing home resident (OR 4.9; 95% CI 1.02–2.5), with no evidence of any benefit in survival among those undergoing or not undergoing assisted feeding. Overall mean survival was 6 months despite hospitalization and the support measures being discussed herein.

The characteristics of the facility where demented patients reside have a similar bearing, showing that the decision to admit a patient to PEG may often be influenced by nonclinical factors. In the study by Mitchell et al., the determining facility characteristics were the presence of a speech therapist, a high number of Medicaid beds, patients aged over 65 years, a limited number of advanced directives issued by patients, over 10% of patients with pressure ulcers, lack of an Alzheimer's unit, and a limited number of full-time nurses with a high number of functionally dependent patients [95–99]. In sum, in the USA, PEG is chiefly adopted in private, urban-based facilities with a higher proportion of Hispanic or black, more elderly, more dependent people, and a disproportionate ratio to staff and care facilities. PEG therefore has the advantage of appearing to be more of an economic-management solution compared to more “natural” options. Spoon feeding takes up a lot of care staff time, and the administrative and financial effects of type of feeding method are part of assessments of appropriateness in the choice of feeding support, particularly for institutionalized patients. The complexity of regulatory and administrative factors in the various nations does not, however, help extrapolate unequivocal trends. As regards PEG-related costs, 29% appear to be due to the procedure, 25% to costs for food formulations, and 33% to hospital costs relating to induced complications [100]. These observations were completed by a cost utility analysis, demonstrating that the cost-effectiveness ratio was positive compared to other forms of feeding support, when the patient was treated for long periods at home. It was instead negative when the patient was a nursing home resident, particularly in healthcare systems where the state is the main contributor to residential care facility costs [101–104].

PEG is apparently preferred by relatives who, in 78% of cases, considered consent to be a moral duty and an extremely stressful choice; in 32% of cases, religious or moral opinions of relatives determined the decision [105]. The majority of relatives did not consider that they had been sufficiently informed on the method and its future clinical impact, since they had not had enough time to reflect on the decision and had been presented with a choice without alternatives. To study how persuaded they were with adoption of the procedure, relatives and care staff were asked if they agreed to suspend assisted feeding after an average period of 17 months. The most frequent response was “I do not know,” with 36% convinced of the need to continue. Some relatives did, however, show remorse about their decision, due in part to assurances they had given to the patient that they would not let them become totally dependent [106]. A decision aid tool containing accurate information on possible options and outcomes with details on consequential processes proved to be very helpful for those family members who were particularly undecided on the choice of artificial support for their relative. This tool tended not to change previously made decisions but proved to be of comfort and support in such emotionally difficult circumstances for those who had to consent to PEG placement in a demented patient [107]. Nonetheless, these decision makers were against PEG placement for themselves in 46% of cases [108].

Options are, however, often influenced by lack of information at various decision-making levels since relatives and carers were unaware of patient preferences, which had been expressed and collected in only 24% of cases. When relatives express the presumed will of the patient, there is a risk of poor objective correlation. In this respect, the outcome of interviews with cognitively intact patients aged over 65, living in different settings, showed that, in the prospect of becoming demented, interviewees ruled out for themselves every form of cardiopulmonary resuscitation, use of artificial respirators, or parenteral or enteral feeding, at all levels of cognitive decline. The factors found to determine acceptance of life-sustaining procedures in the elderly were low schooling level, less autonomy, and a higher perceived quality of life [109]. The quality of preprocedure informed consent was in fact poor, and Brett and Rosenberg [110] demonstrated that, in only one out of 154 analysed cases, consent was given on the basis of adequate discussion of the benefits and secondary effects. In 36% of cases, a patient who was probably competent had signed the consent form. Among the remaining patients, a surrogate decision maker had given consent in 21 cases out of 33, 24% of consents not given by patients were obtained over the telephone with a nurse who signed the document, even though PEG is always electively inserted, and the consenting relatives often lived close to the hospital and therefore had plenty of opportunities for an informative decision-making interview with the attending providers [111].

In a study reporting a 30-day mortality rate of 14.5%, according to an interview conducted after PEG placement in a family member, relatives felt that their consent had contributed to improving the patient's quality of life, although consent was considered to have been formal in only 93% of cases. Relatives are not informed about the implications of PEG placement considering that 28% of them reported not having had a sufficiently informative interview about the PEG system, having been unaware of the complications (46%) and having believed it would help reduce care time. Evidence has shown that PEG feeding reduces caregiver care burden compared to nasogastric tube, and even more so to oral feeding. Orally feeding disabled elderly is undoubtedly difficult, time consuming, and challenging for the ordinary caregiver. The question remains open as to whether these needs are enough to justify the consequences of PEG placement [112]. Nonetheless, the presence of spouses does tend to be the most influential factor in the request for tube feeding.

The characteristics of referring doctors have also been studied. The providers referring older patients for tube feeding were found to be prevalently young internists without specific training on the tube feeding technique, concerned about medical-legal issues, and emotionally involved. One Japanese study in particular, based on a qualitative analysis of physician decisions, showed the determinants to be the health reimbursement system which permits institutionalized long-stay patients; legal barriers to treatment withdrawal; the idea of inducing death by “starvation”; the cultural values of the family responsible for making end of life decisions for the patient; lastly, economic benefits relating to reimbursement of the adopted technique [113].

As concerns racial differences in patients undergoing PEG, the difference in attitude of white and black doctors vis-à-vis end-of-life decisions and assisted feeding has also been studied. Racial differences in orientation have been confirmed among doctors, too. Black doctors seem more inclined to accept aggressive interventions for themselves, and this seems to rule out the aforementioned role of socioeconomic status in racial differences [114, 115]. There is also evidence that 10% of consultants would propose PEG for a demented patient compared to 31% of general practitioners and 45% of nurses. This choice was motivated in less than one quarter of healthcare staff by the hope of increasing the life expectancy of these patients. Dieticians are the only health provider group in which the attitude of the majority is in keeping with the most recent guidelines [116]. Clearly the management process surrounding PEG placement is a structured, multidisciplinary pathway based on the need to address (partly unknown) frail-elderly-related malnutrition, present in 10% of all institutionalized people and underlying the risk of complications and longer hospital stays [117].

Intervening to reduce the improper use of tube feeding in hospital settings, where not all providers are attentive to and constantly updated on developments in evidence on assisted feeding in demented patients, is a complex process that implies structured quality improvement methods. In one UK experience, a significant reduction in improper placements was obtained by an intensive educational scheme. It was done by involving the palliative care service in medical grand rounds and in-service training sessions to stimulate the expected cultural shift, which was positively accepted by doctors from different specialities at the end of the process [118].

Malnutrition needs to be tackled in its early stages through provision of adequate nutritional support by natural means and correct metabolic-dietary management. Periods of inappropriate fasting need to be limited, with continuous stimulation of oral intake, partly through supplements; attention must also be paid to oral hygiene and the swallowing process [119].

Practical guidelines start from the premise that there is a paucity of evidence on recommendations for tube feeding in demented patients and help avoid improper management “short cuts” for these challenging patients, whose numbers are continually increasing in hospitals. Undoubtedly the best management support is given by hospitals with structured assisted feeding units dedicated to these patients.

When a patient is referred for PEG placement, it is essential to establish whether the dysphagia is prevalently acute or chronic, with reference to the patient's general residual functional capacity over time. If the dysphagia is the result of acute decompensation, the decompensation should be treated and an assessment made of whether the patient's management environment can cope with the elderly person's problems, in relation to any advanced directives, or is influencing any behaviours leading to refusal to eat. Drugs that influence swallowing capacity include antipsychotic agents (due to the dystonia they can induce), antidepressants, and benzodiazepines. Intervening in depression and mood can, however, foster an improved relationship with food in demented patients.

The evidence provided thus far gives some insight into the difference between early invasive approaches in elderly demented patients and management of severe swallowing problems. In agreement with the indications cited herein, this may be based on attentive structured assisted feeding programmes, (temporary) artificial hydration, (temporary) slim nasogastric tubes, PEG, or avoidance of feeding. It is therefore essential to carefully consider the prognosis of these patients when deciding treatment. The fact that they are referred to a specialist hospital service, such as endoscopy, could provide an opportunity for planning care and adequate assistance including, for example, adoption of a solely palliative approach. This option would start a pathway to ensure the physical, psychological, social, and spiritual well-being of patients and their families, with a view to guaranteeing maintenance of dignity until the end [120].

PEG remains an important form of support for patients who are stabilized, are not terminal, and who can be shown to benefit from a preliminary period of tube feeding, where there are any doubts. Enteral nutrition in keeping with ESPEN guidelines serves to maintain and improve nutritional status, preserving and improving patient functionality, activity, and capacity for rehabilitation, thereby enhancing quality of life, with the main purpose of reducing morbidity and mortality. The metabolic consequences of aging, which can lead to sarcopenia and alterations in patients' metabolic state, can hinder the success of nutritional therapy.

Early intervention is recommended for unintended weight loss >5% in 3 months or >19% in 6 months, BMI <20 kg/m^2^. Tube feeding is helpful in some neurological disorders and in overcoming depressive anorexia, while making sure in all cases that artificial feeding support is consistent with maintenance of residual functional capacity and not reduction of mobility. In any event, what increases mean survival is maintenance of oral feeding [121], even with dietary supplements, and the frail elderly person can benefit from tube feeding until his/her conditions are stable and not in the terminal stage of a disease [122].

This paper provides a narrative rather than a systematic appraisal of the literature, hinged on the view that there is a lack of robust evidence for the best clinical options for dysphagic elderly with dementia. We have followed the structure of the 2009 Cochrane review on enteral tube feeding for older people with advanced dementia [123] and sought to provide management and decision-making elements for those, like endoscopists, not directly involved in the clinical management of these patients, but who can help optimize patient handling by providing not only their technical expertise but also advice. Moreover, the 2009 review concludes by stating that despite the wide spread of assisted tube feeding, there is insufficient evidence on its actual efficacy in influencing “survival, quality of life, nutrition and pressure ulcers.” Hence endoscopists should at least actively observe the principle, *primum non nocere*.
